# Development of the PEA-PODS (Perceptions of the Environment and Patterns of Diet at School) Survey for Students

**DOI:** 10.5888/pcd15.170561

**Published:** 2018-06-28

**Authors:** Hannah G. Lane, Rebecca Driessen, Katherine Campbell, Rachel Deitch, Lindsey Turner, Elizabeth A. Parker, Erin R. Hager

**Affiliations:** 1 University of Maryland School of Medicine, Department of Pediatrics, Division of Growth and Nutrition; 2 Creighton University School of Medicine; 3 Boise State University, Initiative for Healthy Schools, College of Education; 4 University of Maryland School of Medicine, Department of Family and Community Medicine, Center for Integrative Medicine

## Abstract

**Introduction:**

Few instruments assess key outcomes of school-based obesity interventions, including student perceptions of school environments and school-specific dietary intake patterns. This study describes development of PEA-PODS (Perceptions of the Environment and Patterns of Diet at School), a 2-part survey to measure these outcomes.

**Methods:**

Part 1 (PEA) assessed student perceptions of policies, physical environment, and practices related to healthy eating and physical activity at school. Part 2 (PODS) assessed usual intake (ie, frequency, location obtained, and foods consumed) of breakfast and lunch. Foods consumed were presented by MyPlate categories (eg, Fruits, Grains). Students in grades 3, 6, and 9 participated in 2 phases: cognitive pre-testing (n = 10) and reliability/validation testing (n = 58). Both surveys were administered 1 week apart to assess test-retest reliability and 5-day food records validated PODS. Analyses included percent agreement (70% = acceptable), Pearson correlations, and Cronbach α.

**Results:**

Cognitive pre-testing provided feedback on content, length, and age-appropriateness. Percent agreements were acceptable for test-retest reliability of PEA (71%–96%). The final version included 34 items with Likert-type responses in 4 subscales (α ≥0.78). For PODS, agreement for breakfast and lunch location was ≥75% for both reliability and validation. For foods consumed at breakfast, reliability agreement ranged from 74% to 93%, and validation agreement from 68% to 91%. For foods consumed at lunch, agreement ranges were 76% to 95% and 73% to 88%, respectively.

**Conclusion:**

Both parts of the instrument demonstrate acceptable reliability, and PODS demonstrates acceptable validity. This demonstrates appropriateness for assessing perceptions of the environment and usual dietary intake patterns for school-based obesity prevention initiatives.

## Introduction

Schools are a crucial setting for childhood obesity prevention efforts (1,2). Children spend much of their day at school, and schools strongly influence attitudes and behaviors related to healthy eating and physical activity (PA) (3,4). Federally mandated, district-level Local Wellness Policies (LWPs) guide nutrition and PA policies and practices in schools to enable these behaviors (5,6). The degree of LWP implementation in schools affects their impact; thus, it is important to evaluate both school-level implementation and student-level impact (7,8).

LWP implementation and schools’ policies and practices for healthy eating and PA are commonly assessed through single-reporter administrator surveys and interviews (7,9). This method introduces a high likelihood of bias and does not capture the students’ perceptions, which recent qualitative studies suggest are as meaningful as the policies and practices themselves for improving behavioral and weight outcomes (10–13). Brief, psychometrically sound measures are needed to quantify student perceptions and understand these outcomes.

In addition to understanding student perceptions, instruments are needed to assess the impact of LWP implementation on student behaviors. Objective assessment of school-day PA (eg, via accelerometry) and weight status is possible; however, no such objective measure exists for diet. Although several validated self-report questionnaires assess diet behaviors in schools, they focus on specific food groups (eg, fruits, vegetables, and beverages) or assess total energy intake and macronutrients (14). The limitations of such measures for children and adolescents are well described in the literature, including a limited ability to recall general patterns within a specific timeframe (14,15). To determine how LWPs influence students’ dietary behaviors during school, an easy-to-understand tool is needed to assess usual dietary patterns, rather than intake volume, across common food categories.

This article describes development of PEA-PODS (Perceptions of the Environment and Patterns of Diet at School). PEA assesses students’ perceptions of policies, practices, and environment for healthy eating and PA, and PODS assesses students’ usual diet at school, including frequency, location, and type of food consumed. Test-retest reliability of PEA-PODS and validity testing of PODS are reported.

## Methods

Survey development was an iterative, 3-stage process: development, cognitive pretesting, and reliability/validation. PEA-PODS was developed by a team of researchers with expertise in nutrition and school wellness, and evaluated among students in grades 3, 6, and 9. Study procedures were approved by the University of Maryland, Baltimore institutional review board.

### Stage 1: Development

#### Section 1 (PEA)

We generated a question bank based on LWP requirements and instruments used in previous school-based studies (5,7,9). The question bank items asked students about policies and practices in their overall school, classrooms, cafeteria, and at recess. Items consisted of 5-option Likert-type response sets (“never” to “always” or “totally disagree” to “totally agree”).

No gold standards exist for constructs of student perceptions; however, face validity was established by distributing the initial question bank to a nationwide panel of 10 school wellness researchers who provided feedback and contributed additional items. The final question bank consisted of 47 items (Table 1).

**Table 1:** Test-Retest Reliability of Items in PEA (Perceptions of the Environment) Question Bank, Including Subscales and Modifications

Subscale	Item	Most of the Time/Always % (Time 1)	Agreement % a
School physical activity and nutrition wellness policies and practices (n = 11) Cronbach α = 0.78 Test-retest Pearson r = 0.84 (*P* < .001)	If I get to school before school starts, there are places I can be physically active (like playgrounds or basketball courts).	31.6	78.9
	If I stay after school, there are places I can be physically active (like playgrounds or basketball courts).	53.4	82.8
	I like Physical Education (gym) class.	62.1	91.2
	When my class has parties or celebrations during the school day, we get to eat things like candy, chips, cupcakes, and dessert.	36.2	82.5
	I can easily get water at my school when I am thirsty.	75.9	80.7
	I can easily get a sugary drink or non-diet soda at my school when I want one.	6.9	86.2
	We do physical activity (movement and/or stretching) during our school announcements.^b^	24.1	71.9
	My school's announcements include messages about eating healthy foods and being physically active.^b^	8.6	82.5
	I like the taste of the water at my school.^b^	48.3	77.6
	I see signs or posters with pictures of healthy food.^b^	47.4	77.2
	I see signs or posters showing ways to be physically active.	New question	–
Perception of teacher/classroom wellness policies and practices (n = 16) Cronbach α = 0.79; Test-retest Pearson r = 0.80 (*P* < .001)	Teachers at my school give us short breaks in class where we stand up or get out of our seats to move (like brain breaks or energizers).	14.0	91.1
	Teachers at my school have students run laps, do push-ups or another physical activity when someone misbehaves in class.^b^	7.1	92.7
	Teachers at my school give us extra physical activity time for being well behaved in class.^b^	13.8	82.5
	Teachers at my school let us drink water in class.	72.4	81.0
	Teachers at my school let us eat snacks in class.	12.1	91.2
	Teachers at my school give us treats (like candy) when we do a good job in class.	12.1	91.4
	Teachers at my school talk about being physically active or playing sports.^b^	10.3	81.0
	Teachers at my school (besides gym teachers) play sports or do physical activity with us during the school day.	3.4	82.8
	Teachers at my school tell us it is important to move and be active.	25.9	84.5
	Teachers at my school tell us it is important to eat healthy foods.	27.6	74.1
	Teachers at my school eat healthy meals or snacks during the school day.^b^	22.4	77.6
	Teachers at my school drink water during the school day.^b^	53.4	77.6
	Teachers at my school drink sugary drinks or non-diet soda during the school day.^b^	20.7	78.9
	Teachers and principals at my school care about making my school a healthier place.	60.3	86.2
	Teachers at my school are good role models for healthy eating.	32.8	77.6
	Teachers at my school are good role models for physical activity.	41.4	75.9
Perceptions of cafeteria wellness policies and practices (n = 7)^c^	The breakfast provided by the school is healthy.	31.6	78.9
	The breakfast provided by the school cafeteria tastes good.^b^^,^ c	45.5	–
	The lunch provided by the school is healthy.	31.0	79.3
	The lunch provided by the school cafeteria tastes good.^b^^,^ c	41.2	–
	My school cafeteria is clean and a nice place to eat.	50.0	86.2
	I have enough time to eat my lunch during my lunch period.^b^	67.2	87.7
	At my school, we get to try new foods (like taste tests in the classroom or cafeteria).	5.2	94.7
Perceptions of recess wellness policies and practices (n = 6)	My class has recess.	39.7	96.4
	When someone misbehaves in class, teachers at my school take away their recess or make them sit out.	29.8	85.7
	Kids are moving around and being active during outdoor recess.	New question	–
	There are lots of things that kids can play with or do during outdoor recess.	New question	–
	Teachers let kids stand still or sit during outdoor recess.	New question	–
	When we have indoor recess, we are not allowed to move around and be active.	New question	–

a 5-item Likert scale response, agreement based on +/−1; sample size range 56–58.

b Item slightly re-worded.

c Skip pattern on several items reduced the sample size to n = 37–39 and precluded scale diagnostics.

#### Section 2 (PODS)

To evaluate “usual” diet patterns during the school week, PODS asked students the frequency with which they usually ate breakfast and lunch acquired from various locations (eg, home, school cafeteria, school but not cafeteria, restaurant, or before-school program) during a usual school week, and inquired about the types of foods consumed within each MyPlate category (Fruits, Vegetables, Protein, Grains, and Dairy) (16). As MyPlate is taught widely in schools, it was perceived to be recognizable to and easily understood by most schoolchildren. PODS defined each category by using MyPlate definitions, and included an example of how to break down common foods (eg, slice of pepperoni pizza) into categories. Students were asked 2 questions about each category at each location they selected: how often the category was consumed using a 5-option Likert response set (never to always) and the type of foods consumed from a list of common items in each category (eg, toast, bread, or bagel). Example questions are listed in Table 2.

**Table 2:** Test-Retest Reliability and Validity Compared with a 5-day Food Record of PODS (Patterns of Diet at School), a Brief Instrument to Assess Location, Frequency, and Type of Foods Consumed at Breakfast and Lunch During a Usual School Week

Location, Frequency, Type	Breakfast				Lunch			
	Test-retest Reliability^a^ (n = 58)		Validity^b^ (n = 56)		Test-retest Reliability^a^ (n = 58)		Validity^b^ (n = 56)	
	Baseline Survey n (%)	% Agreement	Food Record n (%)	% Agreement	Baseline Survey n (%)	% Agreement	Food Record n (%)	% Agreement
Location: During a normal school week, when you eat breakfast/lunch, where do you usually get the foods you eat?								
Home	51 (88)	86	49 (88)	85	33 (57)	81	31 (57)	67
School	2 (4)		4 (7)		15 (26)		10 (19)	
School (not cafeteria)	1 (2)		0 (0)		2 (3)		0 (0)	
Restaurant or store (on the way to school)	1 (2)		0 (0)		0 (0)		0 (0)	
Before- school program	0 (0)		0 (0)		n/a		n/a	
≥2 locations	2 (4)		3 (5)		7 (12)		13 (24)	
Frequency: During a normal school week, how often do you usually eat breakfast/lunch?^c^								
Always (5 days)	47 (81)	81	45 (80)	75	55 (91)	91	56 (100)	95
Most days (4 days)	6 (10)		5 (9)		0 (0)		0 (0)	
Half the time (3 days)	2 (4)		3 (5)		3 (9)		0 (0)	
Some days (2 days)	3 (5)		2 (4)		0 (0)		0 (0)	
Once in a while (1 day)	0 (0)		1 (2)		0 (0)		0 (0)	
MyPlate Categories: During a normal school week, how often do you usually eat fruits for breakfast/lunch?^d^								
Fruits	34 (59)	74	30 (54)	73	56 (97)	90	48 (86)	82
Vegetables^e^	18 (31)	–	13 (23)	–	36 (62)	76	36 (64)	73
Grains	54 (93)	90	52 (93)	89	51 (88)	84	50 (89)	88
Dairy	50 (86)	81	28 (50)	77	45 (78)	86	46 (82)	80
Protein	36 (62)	78	45 (80)	71	49 (85)	84	41 (73)	75
Sweet/salty snacks^e^	15 (26)	–	14 (25)	–	49 (85)	88	43 (77)	82
Spreads and sauces	36 (62)	76	27 (48)	68	45 (78)	90	37 (66)	80
Beverages	55 (95)	93	54 (96)	91	56 (97)	95	51 (91)	88

a n = 57–58.

b Comparison of Time 1 survey results to daily food records (≥ 3 days).

c 5-item Likert scale response, agreement based on +/−1.

d Because of smaller sample sizes within categories, responses were dichotomized to yes/no for analysis.

e Agreement is not reported for items answered by fewer than 20 (~35%) participant.

### Stage 2: Cognitive Pre-testing

A cognitive pre-testing process with a convenience sample of 4 students each in grades 3 and 6 and 3 students in grade 9 attending 6 schools (n = 11; 73% male) assessed feasibility and age-appropriateness of PEA-PODS. Caregivers provided written informed consent. Students provided written assent and received a $10 gift card. Students took notes as they completed the survey, then gave feedback to the research team, in groups by grade.

For PEA, this feedback clarified Likert scales (the “agree” scale was confusing for some questions) and wording of several questions (clarification when students have multiple teachers).

For PODS, the feedback led to minor changes to food items within MyPlate categories, some altered wording, a second example of breaking down meals, more detailed instructions, and a “read aloud” feature. Additionally, 3 non-MyPlate categories were added based on participant feedback: Sweet/Salty Snacks, Sauces/Spreads, and Beverages. Following pre-testing, PODS consisted of a range of 10–175 questions, depending on branching patterns.

### Stage 3: Validity/Reliability study

A separate cohort of students in grades 3, 6, and 9 was recruited to assess test-retest reliability of PEA-PODS and validity of PODS (Figure). To detect a minimum correlation of 0.36 with 80% power, our target sample was 60 students. We chose to enroll a broad age range, selecting 20 students each from grades 3, 6, and 9. Students were recruited through snowball sampling, including emails on university listservs, social media postings in parent groups, and advertisements through partners at various institutions across the state. Students who attended public school and could complete online surveys independently were eligible. Parents and guardians were mailed study packets and signed informed consent. Student participants signed assent and received a $50 gift card.

**Figure:**
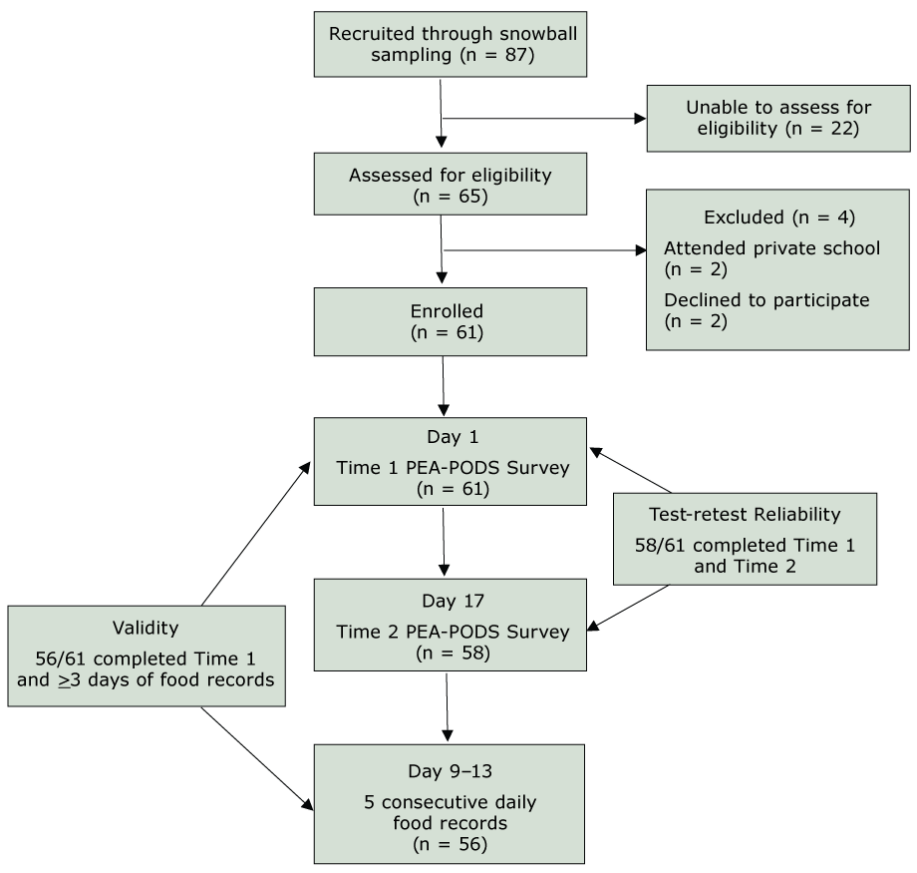
Validation study recruitment, enrollment, and study timeline. Abbreviations: PEA, Perceptions of the Environment; PODS, Patterns of Diet at School.

PEA-PODS was administered electronically by using Qualtrics Version 2017.11 (Provo, UT) on 2 occasions, 1 week apart (Time 1 and Time 2; Figure). The study took place across a period of 2 usual school weeks (without a designated holiday, teacher workday, or early dismissal). To assess test-retest reliability of PEA-PODS, students were emailed survey links on a weekend day, and asked to think about a “usual” school week in the current school year. To assess validity of PODS, participants completed a daily food record on a smartphone by using MovisensXS App Version 1.1. (Karlsruhe, Germany) after Time 2. The daily record is a commonly used method to validate school-based dietary instruments (14). Students were given a basic Android phone with the application pre-loaded and all other features disabled, and were instructed to fill out the food record once daily between 3:00 pm and 11:59 pm for 5 consecutive school days. The app used wording similar to the PODS survey to assess how often students ate breakfast and lunch, where they acquired each, and what type of foods they consumed.

### Data Analysis

Descriptive data were collected from participants during the consenting process. All analyses were conducted in SPSS V.22 (IBM Corp).

#### PEA

**Test-Retest Reliability.** Item-by-item percent agreement was used to examine reliability given a tolerance of +/− 1 (eg, if student answered “Always” at Time 1 and “Most days” at Time 2, responses were considered in agreement) to account for potential minor temporal instability (17). Agreement values between 70% and 79% were considered acceptable, 80% to 89% good, and ≥90% excellent (14,18). Items with poor agreement (<60%) or redundancy within subscales were removed or reworded to enhance clarity, and relevant, theoretical subscales were generated from remaining items. Sum scores were calculated for each subscale at Time 1 and Time 2, and Pearson correlations assessed scale test-retest reliability. Internal consistency of the overall scales and subscales was described by using Cronbach α’s.

#### PODS

**Test-Retest reliability.** Percent agreements assessed test-retest reliability of PODS. For location, agreement was examined for where students reported getting breakfast and lunch during the school week. For frequency, agreement was examined for how often students reported eating breakfast and lunch across locations by using a tolerance of +/− 1, as described above.

For consumption, agreement was examined in 2 ways. First, it was examined for consumption of any food within each MyPlate category (a dichotomous variable was created where 1 represented consumption of any items within that category across locations). Second, a continuous “healthy” composite score was created by using the Dietary Guidelines for Americans to assess whether overall eating patterns could be detected reliably (19). Each food item (eg, toast) was scored separately on a scale of 1–4 (very unhealthy to very healthy) by 3 authors with advanced nutrition degrees, who met to reach consensus (19). To prevent overinflation based on quantity of items selected, mean scores for each MyPlate category were summed to a maximum score of 32 (8 categories × 4 possible points per category). When participants reported getting breakfast, lunch, or both from 2 or more locations, the composite score from the most frequently reported location was used instead of combining all locations, to better identify patterns within locations. Pearson correlation and intraclass correlation coefficients (ICC) of this score described reliability of reporting healthy and unhealthy eating patterns.

Owing to branching patterns, students did not necessarily answer every question — if they reported never eating breakfast, they were not asked further questions about breakfast. Questions with fewer than 20 responses (~35% of the sample) were excluded from analysis.

**Convergent validity.** Percent agreement was also used to compare participants’ reports of location, frequency (with a tolerance of +/− 1), and consumption at Time 1 to aggregated food record data captured over 5 days. After calculating the composite score described above, Pearson correlation coefficients and ICCs assessed validity of reporting of healthy and unhealthy eating patterns. Only participants with at least 3 days of food records were included in analyses. Days of data were converted into a proportion of a full school week, then condensed into categories to match the survey (eg, 0.8–1.0 = Always).

## Results

For the validity and reliability study, 87 students were recruited and 65 were screened for eligibility. Sixty-one (93.8%) consented to participate and completed PEA-PODS at Time 1; 58 (95.1%) completed Time 2 and were included in the reliability analysis, and 56 (91.8%) had 3 or more days of food record data and were included in the validity analysis (Figure).

Of 58 participants who completed PEA-PODS at both time points, 20 were in grade 3, 20 were in grade 6, and 18 were in grade 9. Participants were 53% male, 79% white or Caucasian, 2% black or African American, 2% Hispanic or Latino, 7% Asian, and 5% associated with more than 1 race. Participants represented 8 of 24 (33%) state school districts. Students completed the Time 1 and Time 2 surveys in an average of 50.7 (SD = 16.2) and 46.1 (SD = 16.5) minutes, respectively.

### PEA

#### Test-retest reliability

The original survey contained 47 items. After preliminary analysis of the reliability data, 13 items were removed and 6 were added to complete subscales or to balance a diet or PA construct or both (eg, add “*I see signs or posters with pictures of healthy food*” to balance “*I see signs or posters showing ways to be physically active*”). Additionally, some items were reworded slightly to match other items within subscales (eg, “*I see teachers at my school*” changed to “*Teachers at my school*”). Data were re-analyzed to generate the final results, and 40 items within 4 subscales were included in the final survey: 1) Perceptions of School PA and Nutrition Policies and Practices; 2) Perceptions of Teacher and Classroom Policies and Practices; 3) Perceptions of Cafeteria Policies and Practices; and 4) Perceptions of Recess Policies and Practices. Table 1 includes survey items, responses, item-by-item agreement, and Cronbach α’s and test-retest reliability for the first 2 subscales. Because of the addition of new questions and a skip pattern that reduced the sample size for 2 items, scale reliability could not be calculated for the cafeteria or recess subscales.

Overall, percent agreement was acceptable or good for all items (Table 1). Both the School PA and Nutrition Wellness Policies and Practices and the Perception of Teacher and Classroom Wellness Policies and Practices subscales had a high Cronbach α (0.78 and 0.79, respectively) and good test-retest reliability (Pearson r = 0.84, *P* < 0.001; r = 0.80, *P* < 0.001, respectively).

### PODS

#### Test-retest reliability

Table 2 describes agreement findings for PODS. At Time 1, most participants (n = 51; 88%) reported usually getting their breakfast from home, compared with 4% (n = 2) from school and 4% (n = 2) from more than 1 location. Usual lunch locations included home only (n = 33; 57%), school only (n = 15; 26%), alternating between home and school (n = 6; 10%), and alternating between home and a restaurant or store (n = 1; 2%). Percent agreements for breakfast and lunch location were good (86% and 81%). At Time 1, 91% (n = 53) of the sample reported eating breakfast all or most days of a usual school week, and 91% reported usually eating lunch all days. For frequency, percent agreement was good (81%) for breakfast and excellent (91%) for lunch.

Across locations, agreement in reporting of consumption of foods within MyPlate categories ranged from 74% (Fruits) to 93% (Beverages) for breakfast, and from 76% (Vegetables) to 95% (Beverages) for lunch. Average overall healthy composite scores at Time 1 were 14.6/32 (SD = 5.3) for breakfast and 18.5/32 (SD = 3.9) for lunch (Table 3). Pearson r for breakfast and lunch composite scores were 0.65 and 0.75, respectively. ICCs were 0.66 and 0.73. Pearson correlations and ICCs were significant (*P* < 0.01) and acceptable based on previous literature on adolescent diet indices (18,20–22).

**Table 3:** Healthy Composite Score Reliability and Validity from PODS and 5 Daily Food Records (Pearson r and ICC)

Meal	Test-Retest Reliability^a^				Validation^b^		
	Time 1 Mean (SD)	Time 2 Mean (SD)	Pearson r	ICC^c^	Weekly Mean (SD)	Pearson r	ICC^c^
Breakfast healthy score	14.6 (5.3)	14.7 (5.6)	0.65 (*P* < .001)	0.66 (*P* < .001)	13.1 (4.9)	0.49 (*P* < .001)	0.47 (*P* < .001)
Lunch healthy score	18.5 (3.9)	17.5 (4.6)	0.75 (*P* < .001)	0.73 (*P* < .001)	17.5 (4.7)	0.33 (*P* = .013)	0.33 (*P* = .007)

Abbreviations: ICC, intraclass correlation coefficients; SD, standard deviation.

a Some participants had missing data; therefore, reliability for breakfast, n = 55; reliability for lunch, n = 57.

b Some participants had missing data; therefore, validation for breakfast, n = 53; validation for lunch, n = 54.

c Single-measurement, absolute agreement, two-way mixed effects model.

#### Convergent validity

When comparing Time 1 with the aggregated food records, percent agreements were acceptable, good, or excellent for most location and frequency variables (Table 1). For most MyPlate categories, consumption was similar between the Time 1 survey and the food records for both breakfast and lunch, with higher agreement shown for lunch variables (Table 2). Agreement ranged from 68% to 91% for breakfast, and 73% to 88% for lunch.

Average overall healthy composite scores for the aggregated food record were 13.1/32 (SD = 4.9) for breakfast and 17.5/32 (SD = 4.7) for lunch. Pearson correlation coefficients between Time 1 survey and food records were 0.49 for breakfast (*P* < 0.01) and 0.34 (*P* < 0.05) for lunch. ICCs were similar. Both were significant (*P* < 0.01) and acceptable.

## Discussion

As school-level LWP implementation continues to be a recommended strategy to prevent childhood obesity, it is critical to develop robust, comprehensive evaluation instruments to assess implementation and impact (2). This includes the perception of LWP implementation by students and other key stakeholders in addition to student impact measures (ie, weight status, physical activity, diet). This study fills critical gaps in available evaluation tools by developing PEA-PODS, a novel, reliable survey assessing students’ perceptions of the school environment, as well as a feasible, reliable, and valid method to assess students’ usual dietary patterns at school.

PEA represents a novel method to assess students’ perceptions of schools’ health-promoting environment. Students are influenced by their peers, teachers, and structural environments of their schools (2,3,11); thus, understanding these perceptions is critical to explain health-promoting behaviors. Further, students are key stakeholders in LWP implementation as they uniquely understand their school’s environment, and sustainability depends on their knowledge, acceptance, and identification of gaps and opportunities (23,24). Qualitative studies have reported on student stakeholders’ perceptions of various aspects of the social and structural “health-promoting” environments of their school (10,11,13,25). PEA, which is easy to administer and reliable, builds on these studies by allowing for assessment on a larger scale. PEA also has broad generalizability, as it inquires about components of the school environment that are tied to federal policy requirements (5), and was developed with input from school wellness researchers nationwide. Use of this tool as part of a comprehensive evaluation can provide support for LWPs and other efforts to promote environmental changes in schools.

PODS has several unique strengths, in addition to demonstrating similar reliability and validity compared with existing dietary questionnaires administered during the school day (14,26,27). First, it uses MyPlate, a recognizable, focused framework, to help students categorize foods. The use of these categories within meal-specific prompts, which have been shown to improve self-report accuracy, demonstrates the potential of PODS to reduce known limitations of self-report dietary assessment tools for youth (14,15). Second, unlike previous tools, PODS can assess both inter- and intra-individual differences in types of food consumed by location of origin (eg, school, home, store on the way to school), which can provide information on healthfulness of foods offered at school compared with those brought from home. Previous studies using observational “lunchbox audits” have shown that schools meals are more nutritious than those brought from home. PODS provides a method to understand this on broader scale (28,29). Additionally, it can provide information about what is consumed within consistent food categories. Together, findings would provide support for school meal policies, increase student and parent buy-in for eating meals provided at school, and align with the USDA’s goal to ensure access to safe and balanced meals for all children by increasing school meals participation (30). PODS was also reliable and valid in detecting dietary patterns, as determined by a simple composite score. Finally, PEA-PODS was validated for a broad age range (grades 3–9) including adolescents, a population that is often excluded from dietary instrument validation studies (14,15).

### Limitations

Several limitations should be considered when interpreting study findings. Because we added or reworded 18 items in PEA, further psychometric testing is warranted. PODS, similar to other food frequency questionnaires, is potentially prone to self-report errors; however, the reliability and validity of PODS were acceptable compared with other questionnaires, and both PODS and food records had a low participant burden, which is critical for school-based studies. Although PODS was valid and reliable in detecting dietary patterns using a simple composite score, future studies should consider a more sophisticated system and larger sample to identify dietary patterns (eg, factor analysis). Additionally, as PEA-PODS does not account for the influence of parents or the home environment on student perceptions and diet, it should be administered in conjunction with parent surveys or other methods to understand the home environment.

Findings should also be considered within the small, homogenous sample. As our sample size limited analysis of variables that were not answered by at least 50% of the sample, we were unable to further investigate items within MyPlate categories. Further, we could not compare foods accessed from different locations because of insufficient variability. Finally, although our sample included a broad age range and representation from one-third of state school districts, it lacked racial and ethnic diversity.

### Conclusion

This study developed PEA-PODS, 2 distinct yet complementary surveys for examining student perceptions of the school environment and dietary patterns in school, filling a significant gap in the school wellness literature. Each survey may be administered independently. PEA-PODS was found to be reliable, with the dietary component (PODS) also demonstrating validity. Given the focus on LWPs following a recent final rule (6), PEA-PODS is timely and may be used to examine implementation and impact of LWPs in addition to other school health promotion initiatives.
